# Post-COVID-19 Polymyositis: A Case Report

**DOI:** 10.7759/cureus.30991

**Published:** 2022-11-01

**Authors:** Stefan Anthony, Davong D Phrathep, Ali El-Husari, Ameije Ismaili, Kevin D Healey, Randy Scott

**Affiliations:** 1 Family Medicine, Lake Erie College of Osteopathic Medicine, Bradenton, USA

**Keywords:** idiopathic inflammatory myopathy, arthralgia, myalgia, polymyositis, covid-19

## Abstract

Post-Coronavirus disease 2019 (COVID-19) development of polymyositis is rare, with very few cases documented in the literature. In patients developing vague symptoms after recovery from COVID-19, it is important to investigate all avenues, including the possibility of polymyositis. Polymyositis is typically characterized as symmetrical muscle weakness and histological, electrical, and chemical evidence of muscle injury. Patients identified can be treated with immunosuppressants to return some quality of life and prevent disease progression. In this report, we describe a 58-year-old Caucasian male who presented with symptoms of weakness, myalgias, and arthralgias, six months after recovering from flu-like symptoms of COVID-19. The patient was tested for other autoimmune etiologies and myopathies without positive results. He was treated with prednisone and reported moderate improvement in symptoms. Unfortunately, the patient declined a muscle biopsy or electromyographic testing. According to the criteria for polymyositis set by the Myositis Association and the response to therapy, the patient's symptoms pointed to a likely diagnosis of post-COVID-19 polymyositis.

## Introduction

Polymyositis is an idiopathic inflammatory myopathy (IIM) that should be considered in patients with symmetric, proximal muscle weakness [1]. The signs and symptoms of polymyositis typically develop gradually in middle-aged adults, and more women are affected than men. Although the etiology of polymyositis is unknown, current evidence suggests that it is an autoimmune disorder triggered by a virus and/or an inflammatory state [2]. Traditionally, the Bohan and Peter Criteria for Polymyositis was a widely used classification criterion for the diagnosis of IIM; however, it is outdated and carries limitations [3]. To formulate a diagnosis for our patient, the "Diagnostic Criteria for Polymyositis" was used from the "Myositis Association" [4]. Corticosteroids and immunosuppressive agents are the mainstays of therapy for polymyositis [2]. Recent literature has found inflammatory myopathies linked to Coronavirus disease 2019 (COVID-19), with only two reported cases of polymyositis following COVID-19 in the United States [5]. Because of these recent discoveries, we postulate that COVID-19 could be an etiology of polymyositis. Here we report a case of a 58-year-old male with post-COVID-19 polymyositis.

## Case presentation

A 58-year-old Caucasian male patient reported to the outpatient family medicine clinic with complaints of severe muscle weakness, myalgias, and arthralgias, as well as visual symptoms and palpitations that began after contracting COVID-19 six months ago. Initially, the patient experienced a mild flu-like illness due to a COVID-19 infection that resolved with supportive treatment. After these initial symptoms, he described his myalgias as constant muscle fatigue, weakness, soreness, and pain that worsened with repetitive muscle use. These symptoms were diffusely experienced in the upper and lower extremities, neck, back, and gluteal muscles. He also described arthralgias in his neck, lower back, shoulder, elbows, wrists, hips, knees, and ankles. He also reported an intermittent persistent fever. The patient also reported palpitations which he stated might be anxiety-related.

Additionally, he described visual anomalies that are exacerbated by fatigue or heat. These visual anomalies were photopsia, and the patient described them as shimmering in his peripheral vision and spots of green, orange, and blue in his central vision. He also has experienced a persistent cough related to his chronic sinusitis. He reported minimal relief of symptoms with Tylenol. Another provider recently prescribed Baclofen and Meloxicam, which had not resolved his symptoms. A fasting blood draw was performed, and laboratory results are shown in Table 1.

**Table 1:** Patient Blood Laboratory Values

Marker	Patient’s Value	Reference value
Inflammatory and Autoimmune Panel		
Creatine Kinase	274 units/L	44-196 units/L
C reactive protein	1.6 mg/L	<8.0 mg/L
Sed Rate by Modified Westergren	9 mm/h	≤ 20 mm/h
Anti-Nuclear Antibody	Negative	Negative
Rheumatoid Factor	< 14 IU/mL	<14 IU/mL
Cyclic Citrullinated Peptide AB	< 16 units	< 20 units
Complete Metabolic Panel		
Glucose	97 mg/dL	65-99 mg/dL
Urea Nitrogen	16 mg/dL	7-25 mg/dL
Creatinine	0.97 mg/dL	0.7-1.33 mg/dL
Sodium	138 mmol/L	135-146 mmol/L
Potassium	4.4 mmol/L	3.5-5.3 mmol/L
Chloride	105 mmol/L	98-110 mmol/L
Calcium	9.5 mg/dL	8.6-10.3 mg/dL
Total Protein	7.4 g/dL	6.1-8.1 g/dL
Alkaline Phosphotase	96 units/L	35-144 units/L
Aspartate Aminotransferase	24 units/L	10-35 units/L
Alanine Aminotransferase	25 units/L	9-46 units/L
Complete Blood Count		
White Blood Cell Count	8.9 Thousand/uL	3.8-10.8 Thousand/uL
Reb Blood Cell Count	4.81 Million/uL	4.20-5.80 Million/uL
Hemoglobin	15.3 g/dL	13.2-17.1 g/dL
Hemocrit	44.9 %	38.5-50%
Platelet Count	229 Thousand/uL	140-400 Thousand/uL
Absolute Eosinophils	623 cells/uL	15-500 cells/uL

At this time, the chief differential diagnoses included polymyositis due to his elevated CK, myalgias, symmetrical muscle weakness, fever, and arthralgias. Due to his visual complaints, multiple sclerosis was a concern as well. Thus, he was prescribed a high-dose prednisone pack for 10-days, and a brain MRI with and without contrast was ordered. After completing the prednisone prescription, he reported improvement in joint pain and increased energy levels but still was experiencing some symptoms, including his visual anomalies and persistent (but improved) myalgias. After encouragement to get his brain MRI done, he agreed to go to imaging. Brain MRI showed a normal brain and did not identify any demyelinating lesions, tumors, infarct, collections, or mass effects. The patient did not express interest in a referral or obtaining a muscle biopsy to solidify a diagnosis of polymyositis. At this time, an unconfirmed diagnosis of COVID-related polymyositis was made. He planned to continue prednisone therapy due to his improvement of symptoms but did not follow up for his next lab draw.

## Discussion

The diagnosis of polymyositis is made in approximately 5 per 100,000 people worldwide, with an incidence of 1.2 million cases per year [4,6]. The pathogenesis of polymyositis is due to inflammatory cell infiltrates within muscle fibers, including, but not limited to, CD4+ T-cells, CD8+ T-cells, B-cells, dendritic cells, and macrophages [4,6]. The indirect effects of pro-inflammatory cytokines are responsible for the destructive nature of the disease [6]. Current literature suggests that in polymyositis, antigen-mediated cytotoxic T-cells attack muscle fibers that express major histocompatibility complex-1 (MHC-1) [7]. Polymyositis is a challenging diagnosis for clinicians to make because of the lack of cutaneous findings [9]. It is important to note that most polymyositis cases lack cutaneous findings; however, "mechanic hands" rash is a hyperkeratotic occurrence over the finger pads, and the lateral aspect of both hands occurs in a minority of cases [9]. Clinical features of polymyositis include the progressive onset of muscular weakness in the proximal muscle of the upper and lower extremities with or without pain [9]. The presentation may involve the weakness of neck flexors with or without associated myalgia [8]. The pelvic girdle muscles are the most frequently involved [8]. Disease progression involves distal musculature resulting in abnormal fine movements of the extremities [9]. Less common presenting symptoms include constipation and dysphagia due to gastrointestinal involvement, finger discoloration due to Raynaud's phenomenon, symmetrical arthritis due to joint involvement, and exertional dyspnea due to restrictive cardiomyopathy [8]. Cardiac involvement carries a poor prognosis [9]. Constitutional signs and symptoms are morning stiffness, fatigue, anorexia, fever, and weight loss [9]. According to the Myositis Association, current diagnostic guidelines for polymyositis require four of the following to be present (Table 2) [4]. 

**Table 2:** Diagnostic Criteria for Polymyositis With permission to reproduce from The Myositis Association [4] CK: Creatine kinase; LDH: Lactate dehydrogenase; ALT: alanine aminotransferase; SGPT: Serum glutamic pyruvic transaminase; AST: aspartate aminotransferase; SGOT: serum glutamic-oxaloacetic transaminase; EMG: Electromyography; CRP: C-reactive protein; ESR: erythrocyte sedimentation rate.

A diagnosis of polymyositis should be considered for patients presenting with no skin symptoms and four of the following criteria:
Symmetrical muscle weakness in the shoulders/upper arms or hips/upper legs and trunk
Elevation of serum levels of skeletal muscle-associated enzymes: CK, aldolase, LDH, transaminases (ALT/SGPT and AST/SGOT)
Muscle pain on grasping or spontaneous pain
Positive for any of the myositis-specific autoantibodies
Nondestructive arthritis or arthralgias
The triad of muscle-related changes on EMG: Short, small, low amplitude polyphasic motor unit potentials + Fibrillation potentials + Bizarre high-frequency repetitive discharges
Signs of systemic inflammation: Fever or elevated CRP or accelerated ESR
Muscle biopsy findings compatible with inflammatory myositis (endomysial inflammatory cell infiltrate)

Our patient fulfilled five of the required criteria (symmetrical muscle weakness in the shoulders/upper arms or hips/upper legs and trunk; elevated creatinine kinase; muscle pain on grasping and spontaneous muscle pain; nondestructive arthritis and arthralgias; fever), granting a final diagnosis of polymyositis according to The Myositis Association [4]. Several conditions must be excluded before the diagnosis of polymyositis can be made. These conditions include electrolyte-related myopathy, endocrinology disorders (hypo/hyperthyroidism, diabetes mellitus, metabolic syndrome), other autoimmune disorders (dermatomyositis, lupus, scleroderma, multiple sclerosis), steroid-related myopathy, Cushing syndrome, fibromyalgia/polymyalgia rheumatic, amyotrophic lateral sclerosis, Guillain-Barré syndrome and drug-induced myopathy (statins, alcohol, antimetabolites, antimalarial agents, antifungal agents) [8]. Corticosteroids are the first-line treatment of polymyositis. Prednisone is the corticosteroid of choice, which is routinely dosed at 1 mg/kg/day for 1-2 months until creatinine kinase levels return to baseline. Treatment response is determined by improvements in muscle strength and decreased creatinine kinase levels [9]. In addition to corticosteroids, immunosuppressants and intravenous immunoglobulin may also be used as alternative therapy [9]. Patients are encouraged to initiate a supervised graded exercise program early in the disease [10]. Polymyositis is a lifelong disease with a poor prognosis [8]. Roughly 10% of patients diagnosed with polymyositis eventually succumb to their illness [8]. Elderly females, African Americans, and patients with systemic involvement and refractory disease carry the worst prognosis [8].

The connection between viral pathogens and their ability to trigger autoimmune disease is well documented throughout medical literature; however, the precise mechanism between their association is unclear and likely multifaceted. Many studies have reported the findings of autoantibodies in COVID-19-infected individuals [10]. Furthermore, the COVID-19 genome contains 28 human proteins with homologous regions to COVID-19 peptides that could aid in forming autoantibodies [10]. Another potential mechanism for auto-immunogenicity stems from COVID-19-induced inflammation. COVID-19 is notoriously dangerous, not necessarily due to the virus itself but the inflammatory response it induces. COVID-19-induced hyperinflammation stems from the initial tissue injury caused by the virus, stimulating the secretion of pro-inflammatory cytokines, which further recruits inflammatory cells and promotes inflammatory reactants [11,12]. The repetition and exacerbation of this process generate what is popularly referred to as the "cytokine storm" or a systemic inflammatory response termed macrophage activation syndrome or secondary hemophagocytic lymphohistiocytosis [11]. COVID-19 predisposing the body to immune hyperactivity makes it possible to understand how post-COVID-related autoimmune syndromes may occur.

Nine other reported cases of polymyositis and dermatomyositis following COVID-19 infection [5]. Two of these cases were reported in the United States, with the other four and two occurring in Europe and Asia [5]. One study did not comment on their patient's clinical course of treatment; in another, the patient died due to COVID-19 infection. However, in all the other seven cases, corticosteroids were used as treatment [10]. In four of these cases, it was monotherapy. Other immune modulators used included methotrexate, azathioprine, cyclophosphamide, mycophenolate mofetil, and intravenous immunoglobulin. Outside of the unreported treatment and patient death, all seven other cases reported favorable outcomes with immunosuppressive treatments [10].

One necessary discussion in this case presentation is the differentiation between true-polymyositis, post-viral myositis (PVM), or polymyositis-like syndrome. PVM presents as myalgias or rhabdomyolysis, and symptoms are typically localized to the calf muscles but can involve other muscle groups [13]. PVM usually persists for a month and then resolves [13]. These features make it an unlikely diagnosis for our patient. Another possible diagnosis is a polymyositis-like syndrome. Considering the hyperinflammation syndromes seen as a complication of COVID-19, it is a real differential diagnosis in our patient. Polymyositis-like syndrome has been described in patients with coexisting hypothyroidism; however, the polymyositis-like syndrome may often be misdiagnosed and represent nonspecific hypothyroid myopathy [14]. There were no signs of hypothyroidism in this patient. Due to the exclusion of these differentials, the patient was diagnosed with polymyositis.

There are several limitations to this case study. One, no muscle biopsy or electromyography (EMG) was performed due to resource limitations and patient preference. A muscle biopsy showing endomysial inflammatory cell infiltrates (T-cells) would have helped solidify a diagnosis of polymyositis [15]. Furthermore, to this point, an EMG showing the triad of short, small, low-amplitude polyphasic motor unit potentials, fibrillation potentials, and high-frequency repetitive discharges would have helped confirm a diagnosis [15]. Another limitation is that despite appropriate glucocorticoid treatment, symptoms were not resolved. Although a moderate reduction in symptoms was seen, oral prednisone, the first-line treatment for polymyositis, was not wholly effective in resolving the patient's condition. Other potential agents such as immunosuppressants (methotrexate, azathioprine, calcineurin inhibitors, mycophenolate mofetil, cyclophosphamide) were not trialed in our patient, nor were potential biologics such as rituximab and abatacept which have shown promise in treating myopathies [16].

## Conclusions

Post-COVID-19 polymyositis is rare, with only nine other cases reported in the literature. It should be considered as a differential diagnosis to anyone who recovers from coronavirus symptoms and later presents with at least 3 of the following: myalgias, symmetrical muscle weakness, muscle biopsy evidence of myositis, elevated serum levels of muscle-associated enzymes, and an electromyographic triad of myopathy after ruling out other forms of myopathy. Early identification of inflammatory myopathies in patients will allow for a more rapid introduction of immunosuppressive treatments, relieving some symptoms and improving quality of life. Further studies and follow-ups are required to elucidate if infection with COVID-19 affects treatment outcomes or alters the prognosis of post-COVID-19 polymyositis.
